# Ovarian teratoma-associated anti-NMDAR encephalitis: a single-institute series of six patients from China

**DOI:** 10.1007/s00404-020-05861-3

**Published:** 2020-11-20

**Authors:** Minhua Yu, Shanji Li, Jie Cheng, Liche Zhou, Zhou Jiang, Wen Di

**Affiliations:** 1 Department of Obstetrics and Gynecology, Ren Ji Hospital, School of Medicine, Shanghai Jiao Tong University, Shanghai, China; 2 Shanghai Key Laboratory of Gynecology, Ren Ji Hospital, School of Medicine, Shanghai Jiao Tong University, Shanghai, China; 3 State Key Laboratory of Oncogenes and Related Genes, Shanghai Cancer Institute, Ren Ji Hospital, School of Medicine, Shanghai Jiao Tong University, Shanghai, China; 4 Center for Reproductive Medicine, Ren Ji Hospital, School of Medicine, Shanghai Jiao Tong University, Shanghai, China; 5 Shanghai Key Laboratory of Assisted Reproduction and Reproductive Genetics, Shanghai, China; 6 Department of Pathology, Ren Ji Hospital, School of Medicine, Shanghai Jiao Tong University, Shanghai, China; 7 Department of Neurology and Institute of Neurology, Ruijin Hospital, School of Medicine, Shanghai Jiao Tong University, Shanghai, China

**Keywords:** Ovarian teratoma, Anti-NMDAR encephalitis, Surgical removal

## Abstract

**Purpose:**

Ovarian teratoma-associated anti-*N*-methyl-d-aspartate receptor encephalitis is a rare disease with uncertain etiology and pathogenesis. The disorder is severe and rare with a great impact on young adults. This study aimed to improve the awareness of the disease from experience in our single center.

**Methods:**

Between July 2012 and December 2019, six patients with ovarian teratoma-associated anti-*N*-methyl-d-aspartate receptor encephalitis were enrolled in Ren Ji Hospital, School of Medicine, Shanghai Jiao Tong University. All patients’ data like manifestations, laboratory and radiological data, treatment, and follow-up were reviewed.

**Results:**

Typical psychotic symptoms, memory, and consciousness disorders accompanied by seizures were observed in all patients from this study. All six patients showed positive signals in serum and cerebrospinal fluid samples for *N*-methyl-d-aspartate receptor and received immunotherapy. Three patients underwent unilateral oophorocystectomy and the other three underwent unilateral oophorectomy through minimally invasive surgeries, including laparoscopic and single-port laparoscopic surgeries. The median follow-up time 24.5 months (range from 6 to 93 months). No death occurred. Two patients had recurrent psychotic symptoms while the left four patients had no mental symptoms or tumor recurrence during postoperative follow-up.

**Conclusions:**

For patients with clinical manifestations of unexplained acute psychiatric symptoms accompanied by seizures, memory, and consciousness disorders, the possibility of anti-*N*-methyl-d-aspartate receptor encephalitis should be considered. To confirm the diagnosis, examinations of anti-*N*-methyl-d-aspartate receptor antibodies need to be completed as early as possible. Immunotherapy and tumor location should be given in time once the diagnosis is defined. We recommended removing the tumor as soon as possible without concerning whether the patient is in the acute phase or not. The surgical procedure should be decided based on pathology, age, fertility desire, and patients’ requirements and it should be ensured that tumors are completely removed during operation. Postoperative follow-up is particularly important.

## Background

Anti-*N*-methyl-d-aspartate receptor (NMDAR) encephalitis is a kind of autoimmune encephalitis (AE) characterized by a complex neuropsychiatric syndrome and the presence of specific antibodies against the GluN1 subunit of the NMDAR in cerebrospinal fluid (CSF) [1]. Teratoma is one of the common primary tumors causing AE. The ectopic expression of the teratoma is speculated to have an anti-NMDAR antibody, which leads to the disease [2]. Tumor resection and immunotherapy are the basic therapeutic management for anti-NMDAR encephalitis. Nevertheless, these antibodies of AE, targeting the neuronal intracellular antigens, often cause irreversible neuronal damage. The disorder is severe and rare with a great impact on young adults. We are here describing 6 ovarian teratoma-associated anti-NMDAR encephalitis patients treated at our institution and reviewing the literature.

## Methods

We enrolled six patients with confirmed ovarian teratoma-associated anti-NMDAR encephalitis between July 2012 and December 2019, at Ren Ji Hospital, School of Medicine, Shanghai Jiao Tong University. The clinical and imaging findings of the six patients were retrospectively reviewed.

## Results

### Clinical information

The mean age of six patients was 25 ± 2.28 years (range from 21 to 27 years) at the time of symptom onset. Clinical features and laboratory results are described in Table 1. The clinical-stage with clear manifest signs can be summarized in six patients, including prodromal, psychotic, unresponsive, hyperkinetic, and gradual recovery.

**Table 1:** Clinical features and laboratory results of six patients

No	Date onset	Age	Prodrome	Time to psychiatric stage (days)	Psychotic symptom	Cognitive decline	Abnormal movement	Autonomic dysfunction	Seizures	Memory loss	EEG
1	July 2012	21	Fever Headache	8	Delirium, agitation, aggressive behavior, irritability	Yes	Yes (choreic movement)	Yes (urinary incontinence)	Yes	Yes (progressing to recent amnesia)	δ and θ waves
2	November 2013	27	None	–	Apathy, mutism, stupor	Yes	Yes (forceful clenching of the teeth)	Yes (digestive system)	Yes	Yes	Diffused slow waves
3	May 2018	27	Fever	4	Hallucination, aggressive behavior,	Yes	Yes (forceful clenching of the teeth, choreic movement)	Yes (respiratory system)	Yes	Yes	Unable to judge
4	April 2018	25	Fever Headache	9	Panic disorder, hallucination	Yes	Yes (forceful clenching of the teeth)	Yes (uroschesis, respiratory system)	Yes	Yes	Unknown
5	June 2019	24	None	–	Mania, apathy	Yes	Yes ( intermittent ocular deviation)	None	Yes	Yes	Slow waves
6	October 2019	26	None	–	Delirium, mania, irritability	Yes	Yes (choreic movement)	None	Yes	Yes	Slow waves

No	CSF				NMDAR Ab		Brain MRI/CT	Time to tumor diagnosis	Ovarian teratoma		Time to gradual recovery (days)	Recurrence
	Opening pressure (mmH_2_O)	WBC count (*10^6/L)	Protein (mg/L)	Glucose (mmol/L)	Serum	CSF			Side	Size (mm)		
1	250	3	325	3.58	+	+	Nothing abnormal detected	4 months	Left ovary	25*28*27	44	Yes
2	150	1	161	4.35	+	+	Nothing abnormal detected	30 days	Right ovary	10*13*12	47	Yes
3	400	12	205	3.43	+ (1:10)	+ (1:32)	Nothing abnormal detected	26 days	Right ovary	15*16*17	34	No
4	128	28	349	3.1	+ (1:10)	+ (1:32)	Nothing abnormal detected	18 days	Left ovary	9*10*10	43	No
5	95	0	179	4.61	+ (1:100)	+ (1:100)	Nothing abnormal detected	19 days	Right ovary	9.4*9.6*9.7	44	No
6	225	2	225	3.5	+ (1:320)	+ (1:100)	Nothing abnormal detected	5 days	Left ovary	18*21*26	37	No

### Prodromal stage

Three patients (50%) in this study presented with viral-like prodromal symptoms. All three patients had fever (body temperature between 37.5 and 38.5℃) and two of them had headaches meanwhile. The three patients developed psychotic symptoms after a mean period of 7 days.

### Psychotic stage

**Fig. 1:**
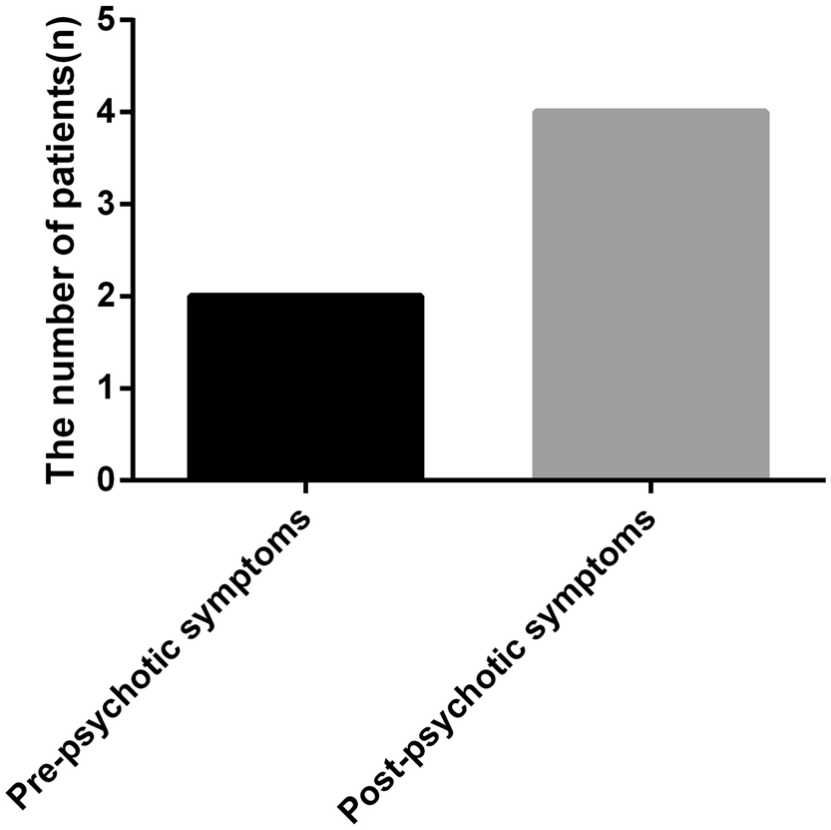
The occurrence of seizures in six patients

All six patients had typical psychotic symptoms at this stage. Initial symptoms included apathy (*n* = 2, 33.33%), delirium (*n* = 2, 33.33%), mania (*n* = 2, 33.33%), irritability (*n* = 2, 33.33%), hallucination (*n* = 2, 33.33%), aggressive behavior (*n* = 2, 33.33%), mutism (*n* = 1, 16.67%), stupor (*n* = 1, 16.67%), agitation(*n* = 1, 16.67%) and panic disorder (*n* = 1, 16.67%). Due to the non-specific symptoms similar to those of acute psychosis, two patients were diagnosed with schizophrenia in the first place. During this period, all six patients had a cognitive decline and could not recall what happened afterwards, with one patient progressing to recent amnesia while follow-up. All patients suffered from epileptic seizures which appeared during the progression of this disease. The initial epileptic seizures of two patients (33.33%) occurred before they had psychotic symptoms and four patients (66.67%) underwent the initial epileptic seizures after psychotic symptoms, shown in Fig. 1.

### Unresponsive stage

Here we observed no clear boundary between the psychotic and unresponsive stage. The six patients developed from the onset to the unresponsive stage after a mean period of 14.83 days (range from 4 to 25 days). Four patients were unresponsive to painful stimuli after psychotic symptoms improving while the other two patients suffered from apathy and other psychotic symptoms as well.

### Hyperkinetic stage

There were abnormal movements and autonomic dysfunction described in the hyperkinetic stage. All six patients showed dyskinesia during the disease, including forceful clenching of the teeth (*n* = 3, 50%), choreic movement (*n* = 3, 50%), and intermittent ocular deviation (*n* = 1, 16.67%). Autonomic dysfunction in the respiratory system was the most common one in this study, affecting two patients. Other symptoms of autonomic dysfunction included urinary incontinence, uroschesis and digestive symptoms.

### Gradual recovery stage

**Fig. 2:**
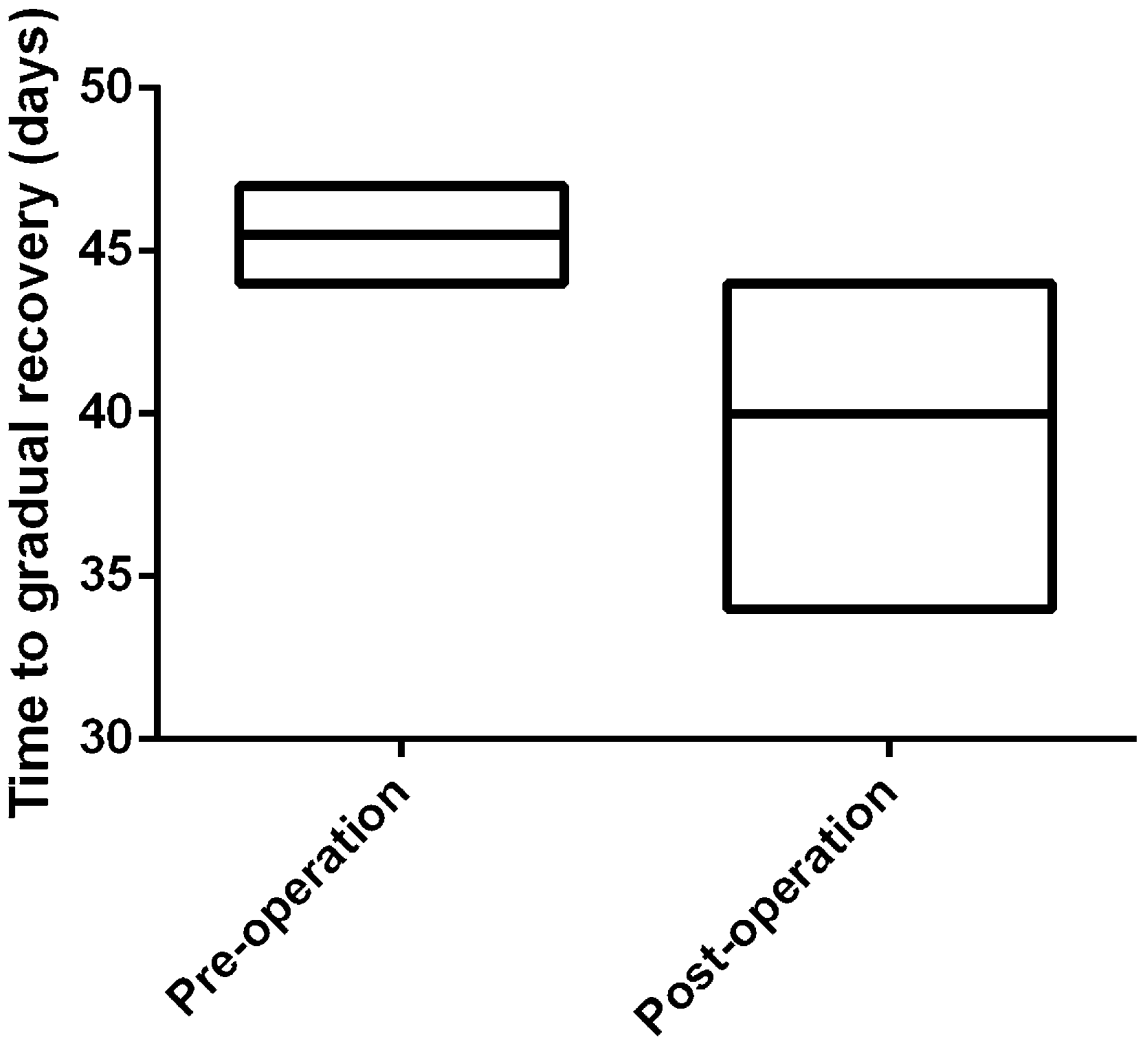
Time to a gradual recovery in two groups

After initial treatment, six patients got gradual recovery after a mean period of 41.5 days (range from 34 to 47 days). Two patients received gynecological operation during their gradual recovery stage and four patients underwent surgeries while they still had obvious symptoms. The mean date from onset to gradual recovery was different in two groups (45.5 days vs 39.5 days respectively), as shown in Fig. 2.

### Laboratory data

Lumbar puncture was performed in all patients after onset. CSF pressure was increased ranging between 225 and 400 mmH_2_O in three patients while normal in the left three ones. Cytological changes in CFS revealed an increased white blood cell (WBC) number in two patients, 12*10^6^/L and 28*10^6^/L respectively. Biochemical examination showed that protein levels were normal and sugar amounts were mostly normal or slightly high. All these patients showed positive signals in serum and CFS samples for NMDAR.

### Electroencephalogram (EEG)

Five patients underwent EEG examination in our institute and one patient had her EEG done in another hospital, which was not recorded in the medical history. Three of them had local or diffused slow waves and one of them had *δ* and *θ* waves. The EEG result of only one patient was unable to be judged due to her stupefaction.

### Head MRI/CT

All six patients received head MRI/CT, which showed no overtly abnormal condition.

#### Ovarian teratoma

**Fig. 3:**
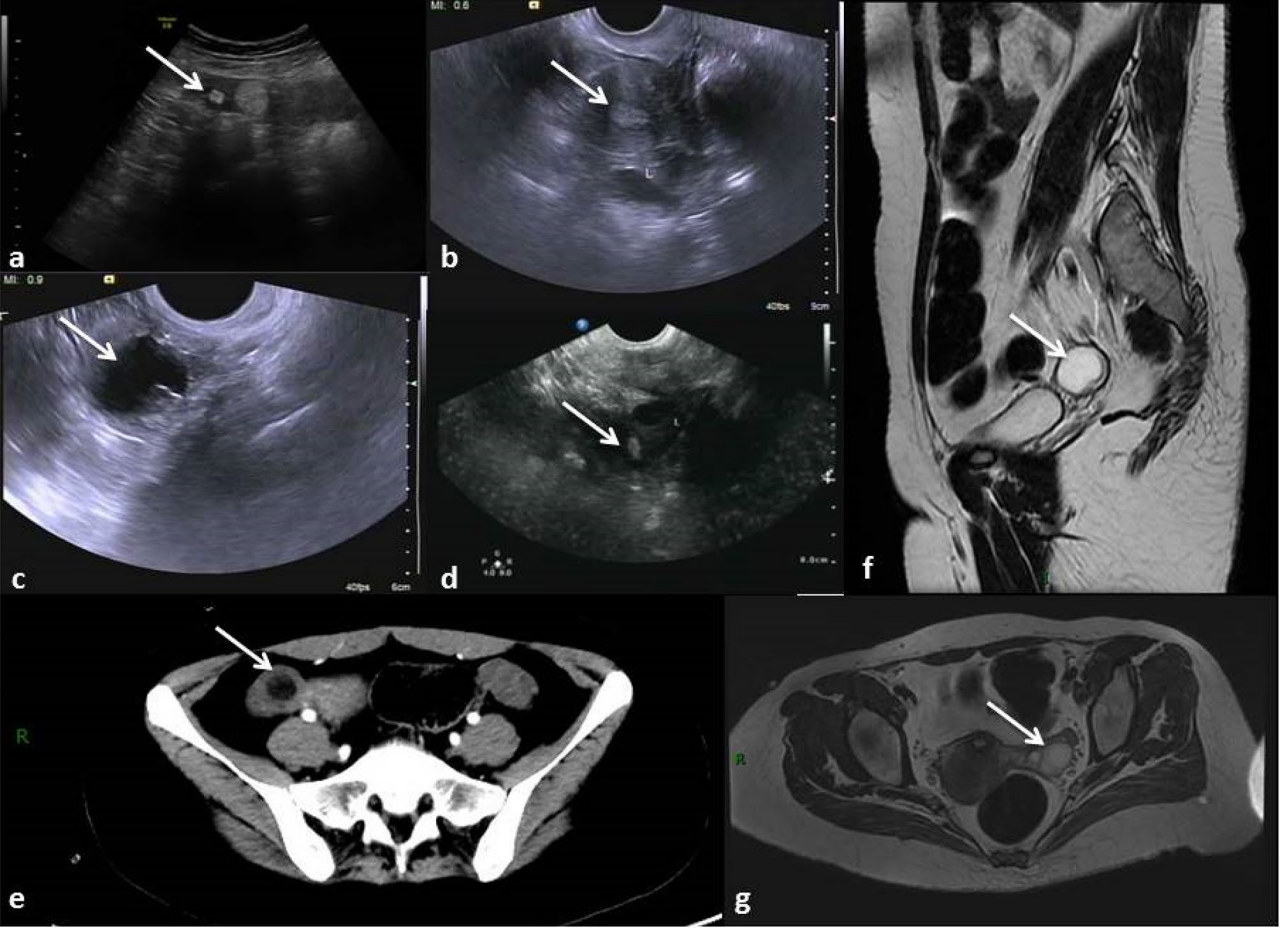
The ovarian cysts detected by ultrasound, CT and MRI for all six patients (arrows); **a**–**d** pictures of ultrasound in four patients (nos. 3–6); **e** CT scan for patient no. 2; **f**, **g**. cross-section and sagittal plane of pelvic MRI for patient no. 1

After positive signals being detected in serum and CFS samples for NMDAR, all six patients were submitted to full-body CT scan and pelvic MRI or ultrasound for tumor screening, shown in Fig. 3. The median date from onset to ovarian teratoma diagnosis was 22.5 days (range from 5 days to 4 months). There was one patient who was not diagnosed with any tumor at the first place through PET-CT and had her ovarian teratoma diagnosed when anti-NMDAR encephalitis recurring, which was 4 months after the initial onset. All six patients had unilateral ovarian cysts, among which three in the right side and three in the left side. Ovarian cysts diameters averaged 1.73 ± 0.80 cm and were confirmed mature teratomas pathologically.

#### Treatment

**Fig. 4:**
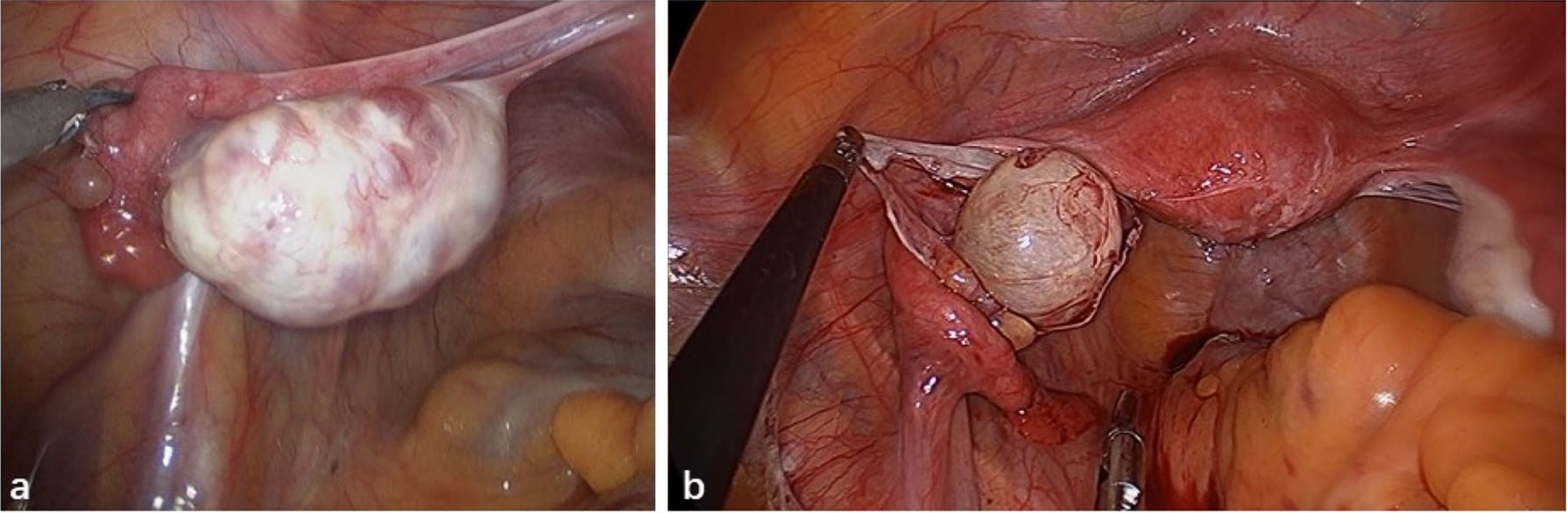
Intraoperative findings of two patients who underwent single-port laparoscopic unilateral oophorectomy (**a**) and unilateral oophorocystectomy (**b**) respectively

Glucocorticoid therapy was administered in all six patients and five of them received first-line immunotherapy with intravenous immunoglobulin (IVIG) therapy. No one underwent plasma exchange. One patients received third-line therapy with mycophenolate mofetil (MMF) when the disease recurring. Other treatments included antibiotics, antiviral drugs, antiepileptic drugs (AED), sedative drugs, and antipsychotic drugs, as shown in Table 2. After the diagnosis of ovarian cysts, three patients underwent unilateral oophorocystectomy and the other three underwent unilateral oophorectomy through minimally invasive surgeries (Fig. 4), including laparoscopic and single-port laparoscopic surgeries. Four of them were sent to the intensive care unit (ICU) immediately after surgeries.

**Table 2:** Treatment for six patients

No	Surgery					Immunotherapy							Others				
						First-line therapy			Second-line therapy		Third-line therapy						
	Time from onset to surgery (days)	Operative pathway	Surgical procedure	ICU stay after surgery	Pathology	Glucocorticoid	IVIG	Plasma enchange	Rituximab	CTX	MMF	Azathioprine	Antibiotics	Antiviral drug	AED	Sedative drug	Antipsychotic drug
1	162	Laparoscopic	Unilateral oophorocystectomy	No	Ovarian mature cystic teratoma	Yes	Yes	No	No	No	Yes	No	Yes	Yes	Yes	Yes	Yes
2	117	Laparoscopic	Unilateral oophorocystectomy	No	Ovarian mature cystic teratoma with mature brain tissue	Yes	No	No	No	No	No	No	No	Yes	Yes	No	Yes
3	33	Single port laparoscopic	Unilateral oophorectomy	Yes	Ovarian mature cystic teratoma	Yes	Yes	No	No	No	No	No	Yes	Yes	Yes	Yes	Yes
4	23	Single port laparoscopic	Unilateral oophorectomy	Yes	Ovarian mature cystic teratoma with mature brain tissue	Yes	Yes	No	No	No	No	No	No	No	Yes	Yes	Yes
5	22	Single port laparoscopic	Unilateral oophorectomy	Yes	Ovarian mature cystic teratoma with mature brain tissue	Yes	Yes	No	No	No	No	No	No	No	Yes	Yes	Yes
6	33	Single port laparoscopic	Unilateral oophorocystectomy	Yes	Ovarian mature cystic teratoma with mature brain tissue	Yes	Yes	No	No	No	No	No	Yes	Yes	Yes	No	Yes

#### Pathology

**Fig. 5:**
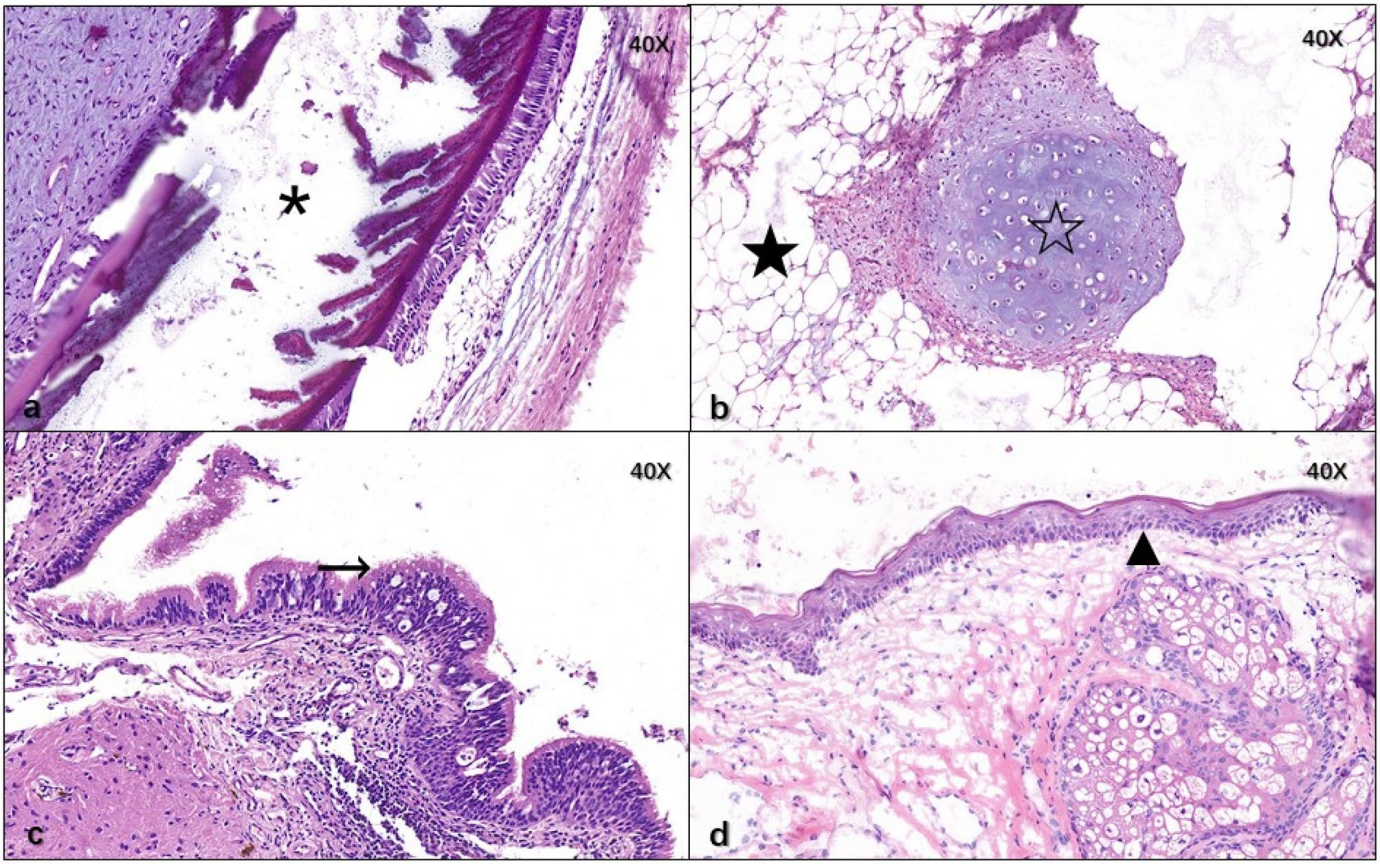
Osseous tissue (asterisk), adipose tissue (filled star), mature cartilage tissue (open star), ciliated columnar epithelium from trachea (right arrow), derma and its appendant (filled triangle) were found in ovarian mature teratoma of the six patients

**Fig. 6:**
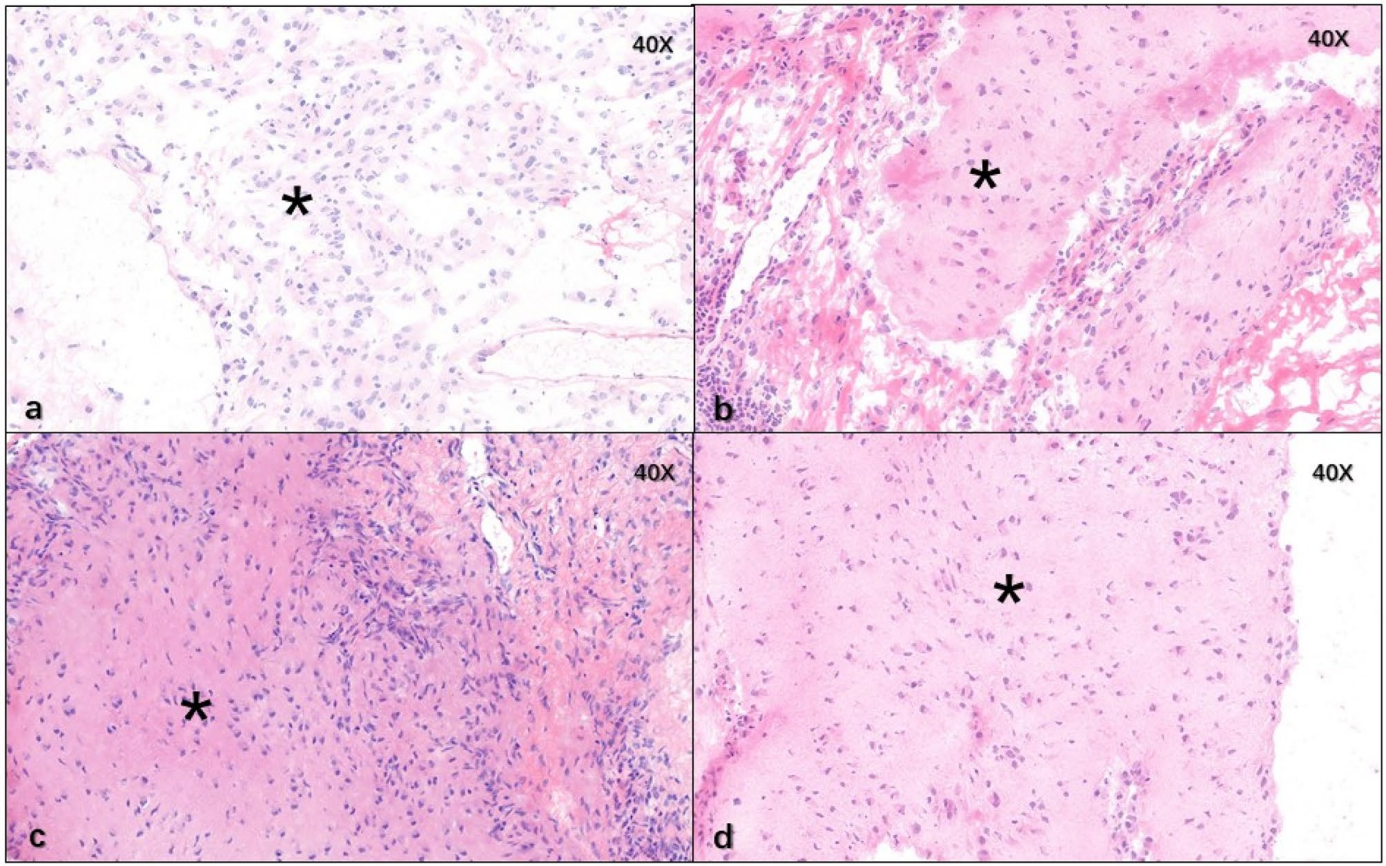
Mature brain tissue (asterisk) was found in pathological sections from patient nos. 2, 4, 5, 6

The ovarian cysts were proven to be mature teratomas pathologically as shown in Fig. 5 and mature brain tissue was found in pathological sections from four patients, shown in Fig. 6.

#### Prognosis

**Fig. 7:**
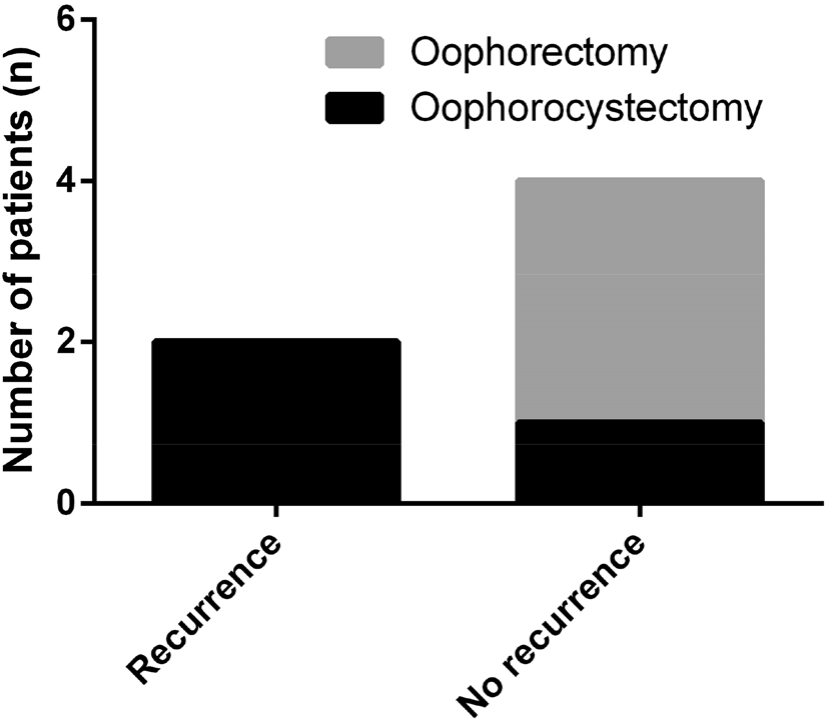
Encephalitis recurrence and different surgery procedures

The median follow-up time of these six patients was 24.5 months (range from 6 to 93 months). There was no death. Four patients who underwent a gynecological operation before they got gradual recovery had no mental symptoms or tumor recurrence during postoperative follow-up. The other two patients who received surgeries during their gradual recovery stage had recurrent psychotic symptoms, of which one patient who was not diagnosed with a tumor at the first place and had her ovarian teratoma diagnosed when anti-NMDAR encephalitis recurring as mentioned above still had recurrent mental symptoms repeatedly after the tumor resection and the other one had encephalitis recurred 8 months after the operation. These two patients were not accompanied with tumor recurrence. By comparison in Fig. 7, patients receiving oophorectomy did not had disease recurrence during follow-up and 66.67% of those who underwent oophorocystectomy (two in three patients) had recurrent psychotic symptoms.

## Discussion

Here we reported a series of six cases of ovarian teratoma-associated anti-NMDAR encephalitis in our hospital. The history of teratoma-associated anti-NMDAR encephalitis can be traced back to 2005.

In 2005, Vitaliani et al. described a syndrome with prominent psychiatric symptoms, memory loss, decreased consciousness and central hypoventilation in four young women with ovarian teratomas [3]. Thereafter, Dalmau et al. identified anti-NMDAR antibodies in serum and CSF, proposing a basic approach in the diagnosis of anti-NMDAR encephalitis in 2007 [4]. Although the disease is rare, with an estimated incidence of 1.5 per million population per year, the impact of this disorder in neurology and psychiatry has been remarkable [5]. In a cohort study of 577 patients with anti-NMDAR encephalitis, 220 patients (38%) had an underlying neoplasm, among which 207 tumors (94%) were ovarian teratomas [6].

The pathogenesis of ovarian teratoma-associated anti-NMDAR encephalitis still maintains unclear. NMDARs are usually formed from heteromers of NR1(which bind glycine) and NR2 subunits (which bind glutamate) [7, 8]. In Dalmau et al.’s study, all examined five tumors contained nervous tissue that strongly expressed NR2 subunits and reacted with patients’ antibodies [4]. Although the mechanisms that trigger the immune response are unclear, scholars postulate the ectopic expression of NR2 subunits by nervous tissue contained in the teratomas contributes to breaking immune tolerance. In our study, mature brain tissue was found in pathological sections from four patients as well, although we did not examine the antigens and antibodies in ovarian teratoma samples from these patients.

Critical roles of NMDARs include synaptic transmission and remodeling, and hippocampal long-term potentiation, one paradigm of memory formation and learning [9]. However, NMDARs are also the major mediator of excitotoxicity, and their dysfunction has been associated with schizophrenia, epilepsy, and several types of dementia. Drugs interacting with NMDARs may result in paranoia, hallucinations, and dyskinesias [10], which contribute to the clinical features of anti-NMDAR encephalitis. The development of anti-NMDAR encephalitis usually follows five stages with distinctive clinical manifestations, as shown in Table 3: (1) Prodromal stage: at this stage, patients usually manifest fever, headache, nausea, vomiting and diarrhea, which are similar to symptoms of viral infection, leading to a misdiagnosis at the first place. The symptoms at prodromal stage maintain for approximately 2 weeks; (2) Psychotic stage: clinical features at this stage mainly are manifest as psychotic symptoms and memory loss, thus this stage can be easily misdiagnosed as schizophrenia or memory impairment; (3) Unresponsive stage: this stage is characterized by dissociated and unresponsive performances. Patients usually refuse to open the eyes, lack of response to painful stimuli, and speaking, but the imitating of language and brain stem reflexes remains normal; (4) Hyperkinetic stage: symptoms at hyperkinetic stage include abnormal movements and dysfunctions of the autonomic nervous system. Abnormal movements mainly refer to abnormal movements of mouth, tongue, and face, dystonia, and dance-like movements. Autonomic dysfunctions are mainly manifested as fever, central ventilation dysfunction, urinary incontinence, uroschesis and digestive symptoms; (5) Gradual recovery stage: this stage is a gradual process with the opposite sequence of symptoms for approximately 3–4 months. Seizures can occur at any stage. In this study, not all patients presented the prodromal stage. Patients at the prodromal stage developed psychotic symptoms after a mean period of 7 days. There are no strict boundaries between psychotic and unresponsive stages, affected by the use of psychotropic drugs as well.

**Table 3:** Five stages of clinical manifestations in anti-NMDAR encephalitis

Five stages of clinical manifestations in anti-NMDAR encephalitis	
Prodromal stage (approximately 2 weeks)	Fever
	Headache
	Nausea
	Vomiting
	Diarrhea
Psychotic stage	Psychotic symptoms
	Memory loss
Unresponsive stage	Dissociated performances
	Unresponsive performances
Hyperkinetic stage	Abnormal movements
	Autonomic dysfunctions
Gradual recovery stage (approximately 3–4 months)	Opposite sequence of symptoms

According to Graus and Titulaer, diagnostic criteria for probable anti-NMDAR encephalitis need to meet all three of the followings: (1) Rapid onset (less 3 months) of at least four of the six following major groups of symptoms—① Abnormal (psychiatric) behavior or cognitive dysfunction; ② Speech dysfunction (pressured speed, verbal reduction, mutism); ③ Movement disorder, dyskinesias, or rigidity/abnormal postures; ④ Decreased level of consciousness; ⑤ Autonomic dysfunction or central hypoventilation; ⑥ Seizures; (2) At least one of the following lab study results—① Abnormal EEG (focal or diffuse slow, epileptic activity or extreme δ brush pattern); ② CSF with pleocytosis or oligoclonal bands; (3) Reasonable exclusion of other disorders. Diagnosis can be made in the presence of one or more of the six major groups of symptoms and IgG anti-GluN1 antibodies after the reasonable exclusion of other disorders. Antibody testing should include CSF. If only serum is available, a confirmatory test should be included (live neurons or tissue immunohistochemistry in addition to cell-based assay) [11]. Therefore, the possibility of anti-NMDAR encephalitis should be considered for young females who present signs of acute abnormal mental behavior, posture and movement disorders, epilepsy, and autonomic dysfunction, which is easily misdiagnosed with schizophrenia, delaying treatment for patients. Due to the fact that part of anti-NMDAR encephalitis is caused by tumors, of which ovarian teratoma is the most common etiology in young females, the full-body radiological examination should be performed for diagnosis. Ovarian teratomas in anti-NMDAR encephalitis are usually not large sizes, and the minimum ovarian teratoma related to this disease as reported worldwide was only 6 mm in diameter [12]. In our study, the ovarian teratoma diameters averaged 1.73 ± 0.80 cm (ranging from 0.9 to 2.8 cm). Since the tumor sizes are sometimes too small to identify, a combination of various imaging methods (ultrasound, CT scan, MRI or PET-CT) are necessary for diagnosis.

Basic therapeutic management for ovarian teratoma-associated anti-NMDAR encephalitis mainly includes tumor resection and immune therapy [13]. From the experience of our institute, the most important treatment is to remove ovarian teratomas as soon as the diagnosis of tumors, even though the risk of surgeries in acute phase of anti-NMDAR encephalitis is high. Patient Nos. 1 and 2 in this study received surgeries during their gradual recovery stage still recurred psychotic symptoms after surgeries while the other four patients who underwent the gynecological operation in the acute phase had no mental symptoms or tumor recurrence during postoperative follow-up, with a shorter period from onset to gradual recovery as well. The antibodies of AE target the neuronal intracellular antigens, often causing irreversible neuronal damage, thus requiring surgeries as soon as possible. The risk of anesthesia during the perioperative period should be of great concern. Three points should be addressed for anesthesia in anti-NMDAR encephalitis: the choice of anesthetic and anesthesia type, appropriate anesthesia maintenance, and postoperative care. Ideally, anesthesiologists should choose the optimal anesthetic for a patient with anti-NMDAR encephalitis, because most anesthetics have some effect on the NMDAR, which may influence the postoperative status. Anesthesia type is also important because it will affect the dose of anesthetic. Unanticipated adverse effects of anesthesia can worsen postoperative status, including delayed awakening from anesthesia, confusion, convulsions, apnea and hypoventilation after extubation, need for tracheal reintubation, aspiration and aspiration pneumonia, among others [14]. Four patients received surgeries during the acute phase of disease were all sent to ICU for postoperative care. Multidisciplinary treatment, including departments of neurology, gynecology, and obstetrics, anesthesiology, and ICU, is particularly important in the treatment of anti-NMDAR encephalitis. Furthermore, for most young women, considering the fertility requirements and protection of the ovary, avoiding residual disease is vital to the surgery. It is currently unclear which of the two surgical procedures, oophorocystectomy, and oophorectomy, is more suitable for patients with ovarian teratoma-associated anti-NMDAR encephalitis. The ovarian cysts were proven to be mature teratomas pathologically in these six patients from our study and patients receiving oophorectomy did not have disease recurrence during follow-up while 66.67% of those who underwent oophorocystectomy (two in three patients) had recurrent psychotic symptoms. It may be the possible reason that the removal of hidden teratoma components containing nerve tissue through oophorectomy. The incidence of anti-NMDAR encephalitis with benign teratoma was 73.9%, with malignant tumor was 21.6%, benign and malignant tissues coexisted was 4.5% as reported [15]. However, the surgical procedure should also be decided considering age, fertility desire and requirements from patients with benign teratomas. Since ovarian teratomas in anti-NMDAR encephalitis are relatively small, it should be ensured that tumors are completely removed during oophorocystectomy. Assistants like intraoperative ultrasound positioning are always helpful and necessary. Laparoscopic surgery should be conducted to prevent leakage of cyst fluid as well.

The prognosis of anti-NMDAR encephalitis is usually better than other types of paraneoplastic encephalitis [16]. Dalmau et al. reported that approximately 75% of patients recovered completely or only suffered minor disabilities [1]. However, the long-term recurrence after surgical excision of mature cystic teratomas is 4.2% as reported, and a patient with young age (< 30 years old) or large cyst (≥ 8 cm in diameter) or bilateral cysts or greater central nervous system component expression rate is at high risk of recurrence, which is even higher when a patient has more than one of these factors [17, 18]. Due to the fact that most patients with ovarian teratoma-associated anti-NMDAR encephalitis are young females and mature brain tissues can be found in pathological sections, postoperative follow-up is particularly important, reexamination every 6 months for at least 4 years is necessary.

## Conclusion

Ovarian teratoma-associated anti-NMDAR encephalitis is a rare disease with uncertain etiology and pathogenesis. Through retrospective analysis of clinical manifestations, laboratory data, and follow-up of six patients in this study, we found that for patients with clinical manifestations of unexplained acute psychiatric symptoms accompanied by seizures, memory and consciousness disorders, the possibility of anti-NMDAR encephalitis should be considered and examinations for anti-NMDAR antibodies need to be completed to confirm the diagnosis as early as possible. Immunotherapy and tumor location should be considered in time once the diagnosis is defined. It is recommended to remove the tumor as soon as possible no matter whether the patient is in the acute phase or not. Multidisciplinary participation can be performed. The choice of surgical procedure should be decided considering pathology, age, fertility desire and patients’ requirements and it should be ensured that tumors are completely removed during operation. Postoperative follow-up is particularly important in case of recurrence.
